# A197 ASSOCIATION BETWEEN ANTIMICROBIAL SEROLOGIES AND IBD LOCATION AND BEHAVIOUR: DATA FROM THE CIDSCANN INCEPTION COHORT

**DOI:** 10.1093/jcag/gwae059.197

**Published:** 2025-02-10

**Authors:** E Fanous Vukas, A Muise, K Jacobson, H Huynh, A Otley, J deBruyn, D Mack, C Deslandres, W El-Matary, T Walters, A Griffiths, A Ricciuto

**Affiliations:** The Hospital for Sick Children, Toronto, ON, Canada; The Hospital for Sick Children, Toronto, ON, Canada; The Hospital for Sick Children, Toronto, ON, Canada; The Hospital for Sick Children, Toronto, ON, Canada; The Hospital for Sick Children, Toronto, ON, Canada; Alberta Children’s Hospital, Calgary, AB, Canada; Children’s Hospital of Eastern Ontario, Ottawa, ON, Canada; Centre Hospitalier Universitaire Sainte-Justine, Montreal, QC, Canada; University of Manitoba Children’s Hospital Research Institute of Manitoba, Winnipeg, MB, Canada; The Hospital for Sick Children, Toronto, ON, Canada; The Hospital for Sick Children, Toronto, ON, Canada; The Hospital for Sick Children, Toronto, ON, Canada

## Abstract

**Background:**

Antimicrobial serologies have been associated with Inflammatory Bowel Disease (IBD) type and Crohn’s disease (CD) progression. However, prospective pediatric data examining these associations while considering disease location are sparse.

**Aims:**

1) Describe rates of serology positivity by disease location; 2) Assess the ability of serologies to predict CD progression to stricturing (B2) /penetrating (B3) behaviour, while accounting for IBD location.

**Methods:**

Children with CD enrolled in the prospective multicentre Canadian Children IBD Network (CIDsCaNN) were included. ASCA IgA and IgG, CBir1, OmpC and ANCA positivity were measured centrally at Cedars-Sinai. We assessed variables at diagnosis and last follow up. We used Pearson’s Chi-Squared test to compare proportions and Mann-Whitney U test to compare medians between groups. We used multivariable Cox regression to determine the association between serologies and progression to B2/B3 amongst CD patients with B1 at diagnosis, while accounting for L1 location. We calculated area under the ROC curve (AUC) to quantify predictive ability.

**Results:**

There were 512 CD patients included: median age 13 y (IQR 10.8-14.9), follow up 3.3 y (IQR 1.9-5.0). There were 50/452 (11%) B1 CD patients who progressed (L1: 16/77 (21%); L2: 7/112 (6%); L3: 27/241 (11%)). ASCA+ was more frequent among patients with L1/L3 compared to L2 CD (28% vs 13%, p=0.009), while CBir1+ and OmpC+ rates did not differ by CD location (55% vs 48%, p=0.19 and 9% vs 9%, p=0.85). ANCA+ rate was higher among L2 CD compared to L1/L3 CD (30% vs 10%, p<0.001). In univariate survival analysis, CBir1+, ASCA+ and L1 were associated with progression to B2/B3 (Table 1). In multivariable analysis, only positivity for CBir1+ and L1 were associated with progression. CBir1 titre had only moderate ability to predict progression (AUC 0.65 (95%CI 0.57-0.72)).

**Conclusions:**

While ASCA and CBir1 are associated with complicated CD in univariate analysis, this appears to be mediated by small bowel location for ASCA. While CBir1 was independently associated with B2/B3 disease, its predictive ability alone was limited.

Table 1. Unadjusted and Adjusted Hazard Ratios for Progression to B2 or B3 Complications

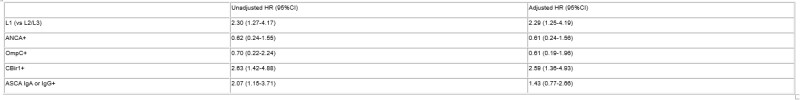

**Funding Agencies:**

CIHRCh.I.L.D Foundation

